# Berberine ameliorates high-fat diet-induced metabolic disorders through promoting gut *Akkermansia* and modulating bile acid metabolism

**DOI:** 10.1186/s13020-025-01251-6

**Published:** 2025-11-17

**Authors:** Wei-jian Hang, Rui Yin, Xi-wei Kang, Lu He, Xuan Cao, Juan Chen

**Affiliations:** 1 Department of Biochemistry and Molecular Biology, School of Basic Medicine and the Collaborative Innovation Center for Brain Science, Tongji Medical College, Huazhong Universi-ty of Science and Technology, Wuhan, 430030 Hubei China; 2 Department of Basic Medicine, School of Medicine, Taizhou University, Taizhou, 318000 Zhejiang China; 3 Faculty of Arts and Science, University of Toronto, Toronto, ON M5S3G3 Canada; 4 Division of Cardiology, Department of Internal Medicine, Tongji Hospital, Tongji Medical College, Huazhong University of Science and Technology, Wuhan, 430060 Hubei China

**Keywords:** Berberine, *Akkermansia*, High fat diet, Metabolomics, Intestinal integrity

## Abstract

**Background:**

*Coptidis Rhizoma*, the rhizome of Coptis chinensis Franch., has long been employed in the treatment of diabetes. Its active component, berberine, has been utilized in clinical practice; however, the underlying mechanisms of its protective effects remain to be fully elucidated.

**Methods:**

Metabolomics and lipidomics analyzed plasma metabolite and lipid changes in mice fed a high-fat diet and treated with 25 mg/kg/day berberine for three months. Metagenomics and microbiota transplantation identified gut microbiota responding to berberine. Co-administration of berberine and *Akkermansia* was studied for metabolic effects, analyzing plasma and fecal metabolomics.

**Results:**

Berberine reduced triglycerides and cholesterol, showing metabolic protective effects. Metagenomics identified *Akkermansia* as key to berberine's benefits, validated by microbiota transplantation. Berberine enhanced *Akkermansia* growth, preserving intestinal mucus and tight junctions. It promotes the conversion of cholesterol to bile acids by inhibiting adenosine 5 ‘-monophosphate -activated protein kinase (AMPK), which promotes the expression of cholesterol 7-alpha hydroxylase (CYP7A1). Co-administration of berberine and *Akkermansia* amplified these effects. Potential metabolites, including linoleic acid and N-acetylputrescine, contributed to the observed benefits.

**Conclusion:**

Berberine, through *Akkermansia*, maintains intestinal integrity and reduces cholesterol, highlighting its potential as a therapeutic agent for metabolic disorders. Combining berberine with *Akkermansia* enhances its efficacy against hyperlipidemia.

**Graphical Abstract:**

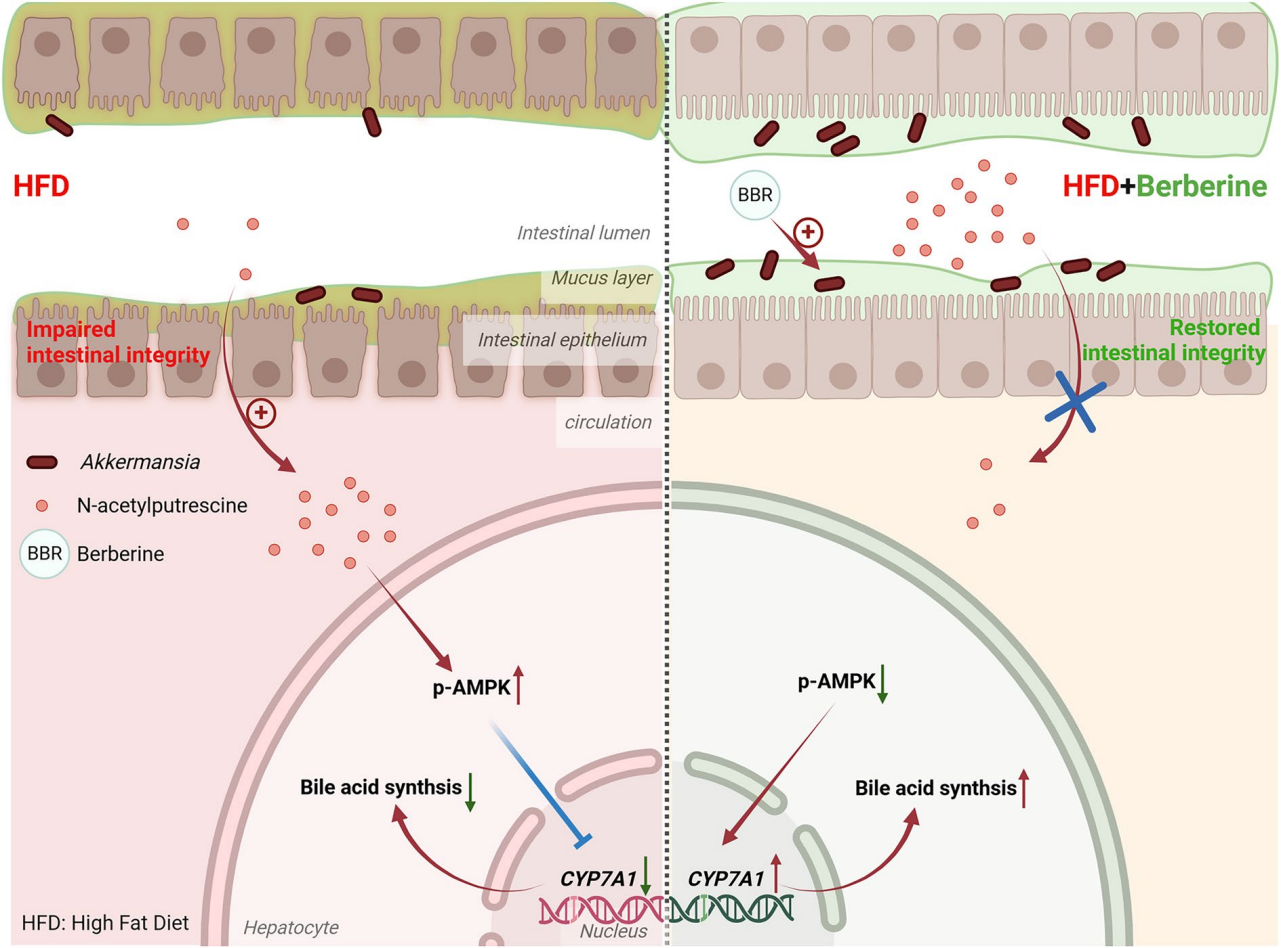

**Supplementary Information:**

The online version contains supplementary material available at 10.1186/s13020-025-01251-6.

## Introduction

Obesity and related metabolic disorders are increasingly significant due to modern lifestyle changes. Obesity, a systemic disease, impacts various organs and elevates the risk of diabetic complications [1]. Blood samples, combined with advanced mass spectrometry, provide comprehensive insights into systemic alterations in conditions like obesity [2].

Traditional Chinese herbs, such as *Coptidis Rhizoma*, are gaining attention for treating metabolic disorders [3]. Its active ingredient, berberine, a natural pentacyclic isoquinoline alkaloid, exhibits notable anti-obesity and anti-diabetic effects, including improved insulin sensitivity and amelioration of diabetic complications [3, 4]. Evidence from clinical, animal, and cellular studies has demonstrated that berberine improves glucose and lipid metabolism in obese individuals [5, 6], but the systemic metabolic changes and key metabolic pathways after berberine administration are still unknown.

Berberine’s bioavailability is notably low (1–5%), yet its protective effects diminish in germ-free or antibiotic-treated mice, indicating a crucial role for gut microbiota [7]. Studies suggest that berberine modulates gut microbiota, affecting systemic metabolism via active metabolites and lipids [8], though the specific microbiota and metabolites remain undefined.

The gut microbiota plays a pivotal role in counteracting obesity, with one of its key mechanisms being the maintenance and reinforcement of intestinal barrier function [9]. A stable microbial ecosystem acts as a “protective shield” for the gut by promoting mucus secretion and enhancing tight junctions between epithelial cells, thereby establishing a robust biological barrier [10]. This barrier not only effectively defends against harmful substances but also ensures proper energy homeostasis and metabolic regulation [11]. Consequently, modulating the gut microbiota to strengthen the intestinal barrier has emerged as an effective strategy in the fight against obesity [12, 13].

High-fat diet (HFD) impair gut microbiota and loosen intestinal tight junctions [14], promoting inflammation through leakage of harmful substrates into circulation [15]. Whether berberine’s effects involve restoring gut barrier integrity is not fully understood. This study used berberine to intervene in HFD-induced obesity in mice, employing multi-omics to elucidate its therapeutic mechanisms.

## Results

### Berberine ameliorates high fat diet induced-metabolic disorder

**Fig. 1:**
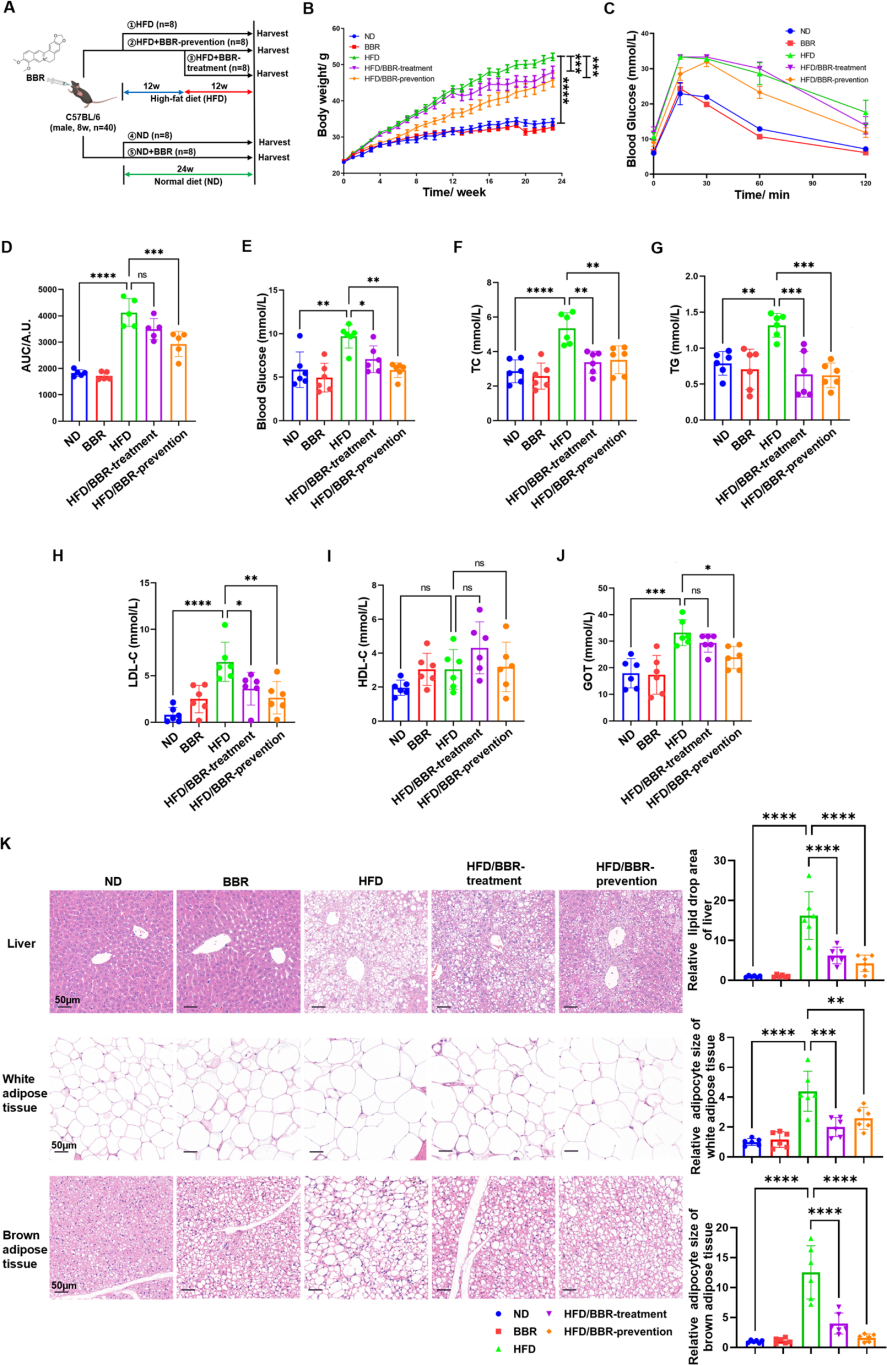
Berberine ameliorates high-fat diet induced metabolic disorder in animal model. **A** animal models with two different time point of berberine intervention. **B** body weight record of mice in different groups. **C** glucose tolerance test of mice in five groups after 24 weeks of HFD and berberine administration. **D** area under curve (AUC) of blood glucose in C, and the statical analysis. **E** plasma concentration of blood glucose in five groups of mice after overnight fasting. **F** plasma concentration of total cholesterol in five groups of mice after overnight fasting. **G** plasma concentration of total triglyceride in five groups of mice after overnight fasting. **H** plasma concentration of LDL-C. **I** plasma concentration of HDL-C in five groups of mice after overnight fasting. **J** plasma concentration of GOT in five groups of mice after overnight fasting. **K** representative image of H&E staining of white adipose tissue and brown adipose tissue and hepatic tissue in five groups. These results are presented as mean ± SD (biological replicates, *n* = 5 for Body weight and GTT assay; *n* = 6 for serum assays). (*P < 0.05, **P < 0.01, ***P < 0.001, ****P < 0.0001, all the data significance was analyzed by ANOVA by Graph Pad Prism Software V.9.0). These experiments were repeated at least three times

Berberine, an active ingredient of *Coptidis Rhizoma* (Fig. S1), was evaluated for its dosage effects in treating high fat diet (HFD)-induced metabolic disorders. A low dosage (10 mg/kg/day) had minimal impact on weight loss, while medium (25 mg/kg/day) and high doses (50 mg/kg/day) showed effects comparable to metformin (100 mg/kg/day) (Fig. S2A-B). Since higher doses did not enhance weight loss, a medium dosage (25 mg/kg/day) was selected for further experiments.C57Bl/6 male mice on a HFD for 24 weeks received berberine at 25 mg/kg/day, starting either at the diet's onset (prevention group) or after 3 months (treatment group) (Fig. 1A). Berberine prevention reduced body weight, and treatment showed similar trends (Fig. 1B). Glucose tolerance tests confirmed improved systemic glucose sensitivity (Fig. 1C-D). Berberine intervention reversed elevated blood glucose, triglycerides, cholesterol, and LDL-C levels (Fig. 1E-H). HDL-C levels remained unchanged (Fig. 1I), but GOT, a hepatic injury marker, was significantly reduced (Fig. 1J). Pathological changes like white adipocyte hypertrophy, brown adipose tissue whitening, and hepatic steatosis were mitigated in both groups (Fig. 1K).

These findings suggest berberine's significant metabolic modulation effects against HFD-induced disorders, providing protection at early and mid-stages.

### Berberine reduces lipid burden and fatty acid metabolism by metabolomics and lipidomics study

**Fig. 2:**
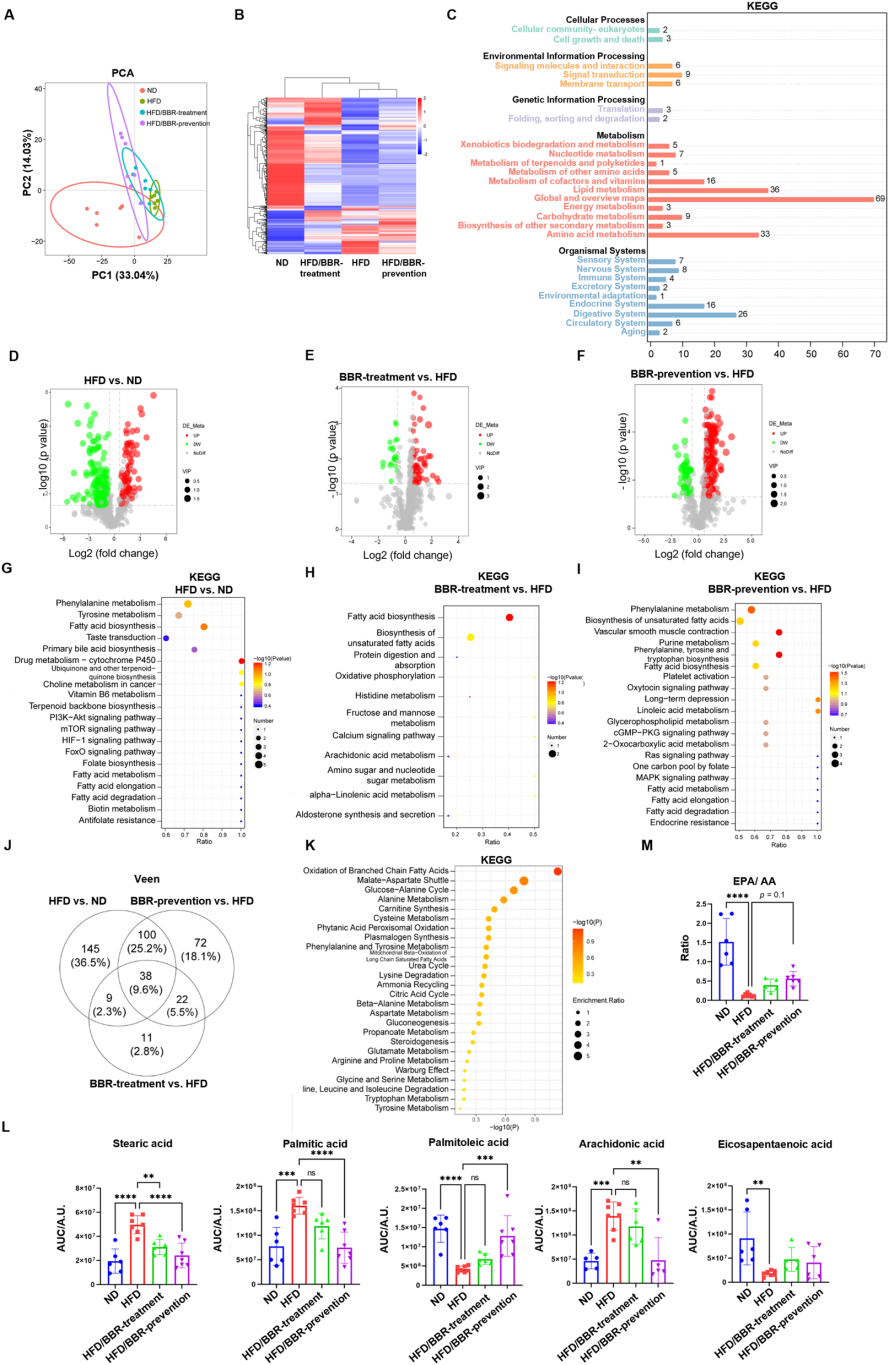
Metabolomics changes in plasma in mice receiving high-fat diet and berberine. **A** PCA analysis of differential expressed metabolites in four groups: normal diet (ND), high-fat diet (HFD), high-fat diet with berberine treatment (HFD/BBR-treatment) and high-fat diet with berberine prevention (HFD/BBR-prevention). **B** heatmap of differential expressed metabolites in four groups. **C** KEGG enrichment of detected differential metabolites. **D** volcano plot of differential expressed metabolites between ND and HFD group. **E** volcano plot of differential expressed metabolites between HFD/BBR-treatment group and HFD group. **F** volcano plot of differential expressed metabolites between HFD/BBR-prevention group and HFD group. **G** KEGG enrichment of differential expressed metabolites between ND and HFD group. **H** KEGG enrichment of differential expressed metabolites between HFD/BBR-treatment group and HFD group. **I** KEGG enrichment of differential expressed metabolites between HFD/BBR-prevention group and HFD group. **J** Veen diagram of differential expressed metabolites in three comparations, indicating those changes of metabolites possibly be affected by berberine. **K** KEGG enrichment of the overlapping 38 metabolites. **L** relative intensity of different unsaturated fatty acids in four groups. **M** the ratio of EPA to AA in four groups. These results are presented as mean ± SD (biological replicates, *n* = 6 for metabolite analysis). (*P < 0.05, **P < 0.01, ***P < 0.001, ****P < 0.0001, all the data significance in figure L and M was analyzed by ANOVA by Graph Pad Prism Software V.9.0). These experiments were repeated at least three times

To investigate metabolic changes in HFD-induced mice, untargeted metabolomics analysis detected 676 metabolites (Fig. 2A&B). PCA and heatmaps showed distinct clustering among groups. KEGG analysis revealed significant enrichment in fatty acid biosynthesis and cytochrome P450 pathways in HFD groups compared to ND (Fig. 2D&G). Berberine treatment primarily affected fatty acid biosynthesis (Fig. 2E&H), while prevention influenced pathways like fatty acid, amino acid, and unsaturated fatty acid metabolism (Fig. 2F&I), highlighting different metabolic alterations based on intervention timing.

Fatty acid metabolism showed significant changes (Fig. 2J&K). Saturated fatty acids (e.g., palmitic acid) were elevated in HFD groups but reduced by berberine treatment or prevention (Fig. 2L). Protective unsaturated fatty acids, such as palmitoleic acid [16], were restored, while detrimental arachidonic acid (AA) levels were decreased (Fig. 2L). EPA/AA ratios, linked to obesity [17], were reduced in HFD mice but improved with berberine prevention (Fig. 2M). These findings indicate berberine reverses metabolic alterations and reduces lipid burden.

**Fig. 3:**
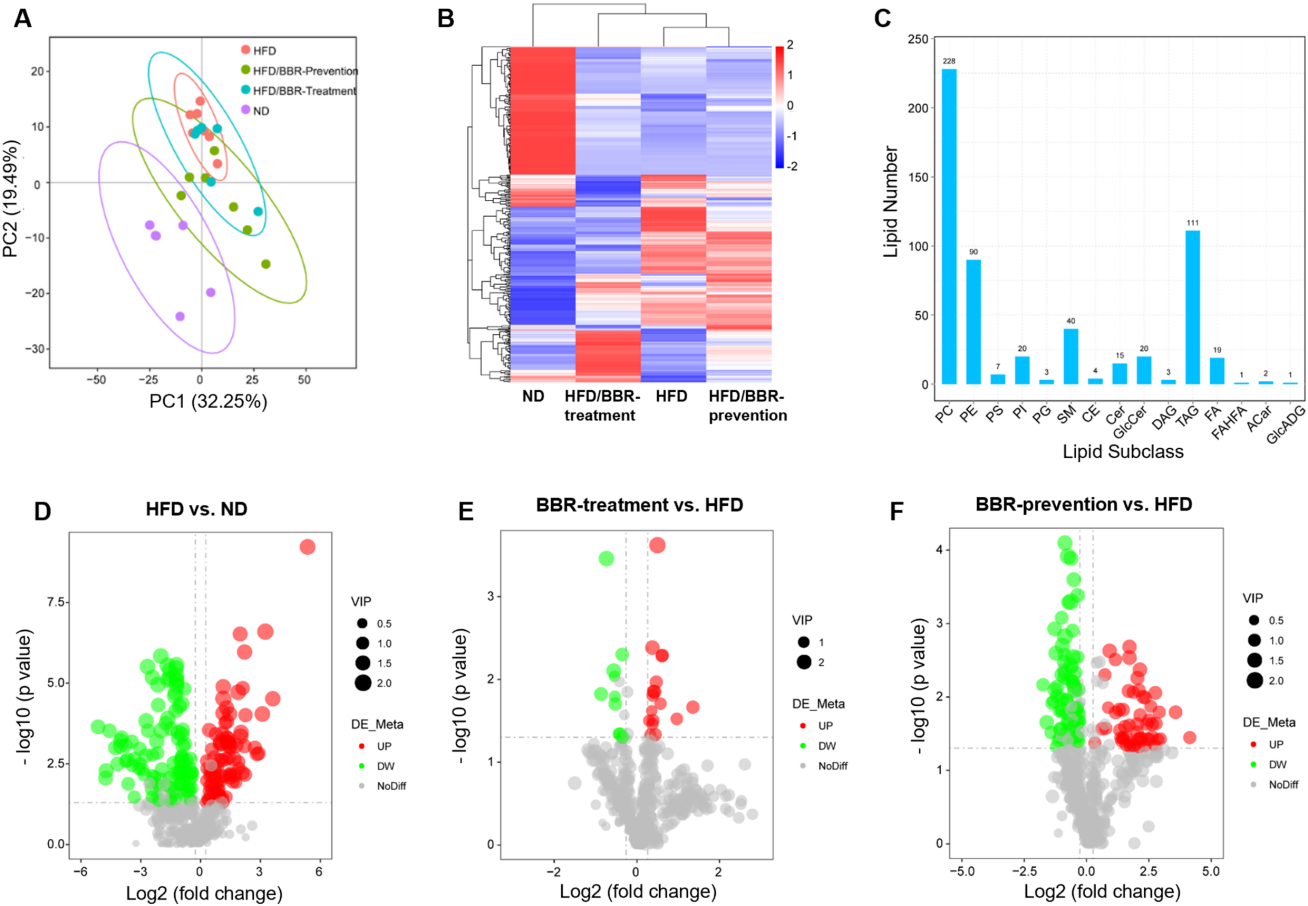
Lipidomics changes in plasma in mice receiving high-fat diet and berberine. **A** PCA cluster of plasma lipids in four groups. **B** heatmap of plasma lipid in mice plasma. **C**. amount of detected differential lipids and their subclass. **D** volcano plot of differential expressed lipids between ND and HFD group. **E** volcano plot of differential expressed lipids between HFD/BBR-treatment group and HFD group. **F** volcano plot of differential expressed lipids between HFD/BBR-prevention group and HFD group

Untargeted lipidomics identified 564 differentially expressed lipids in 15 subclasses, primarily glycerophospholipids and triglycerides (429/564) (Fig. 3A–C, S3). PCA and heatmaps showed the HFD group distinct from ND (Fig. 3A, B). Berberine-prevention groups clustered closer to ND, while treatment groups remained closer to HFD, reflecting the time course of berberine’s effects.

### Berberine alters intestinal microbiota composition and increases the abundance of *Akkermansia*

**Fig. 4:**
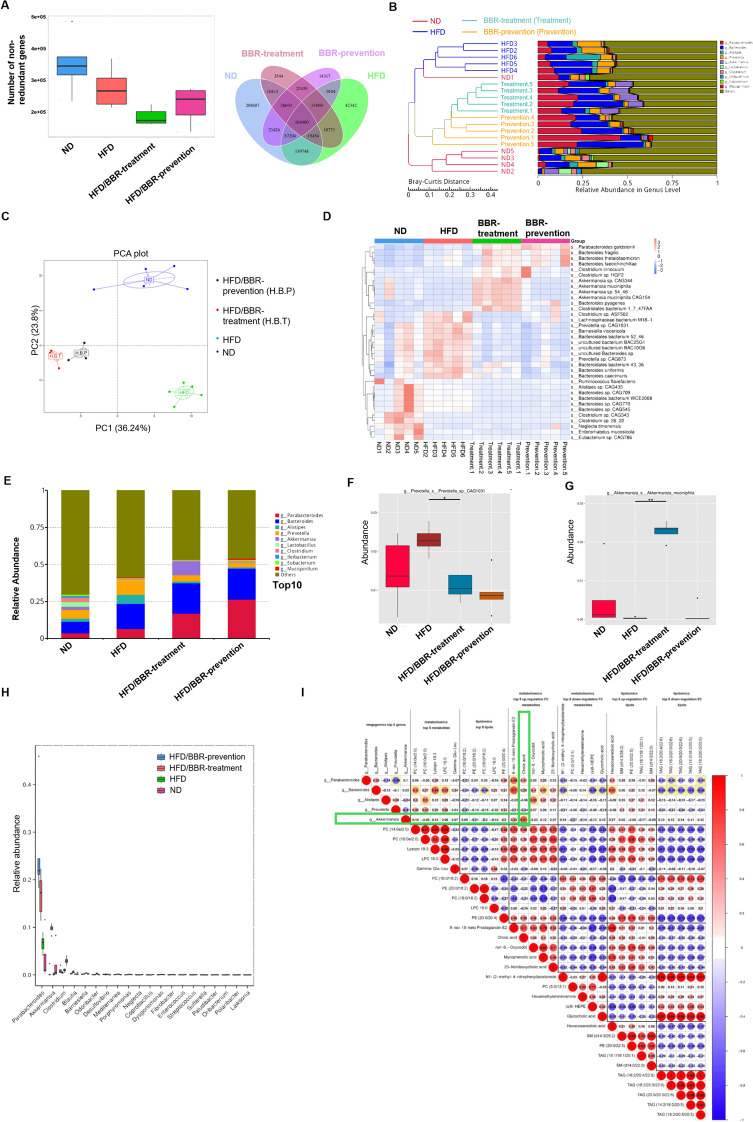
Berberine significantly changes the composition of gut microbiota.** A** unique genes in feces of four groups, and Venn diagram showed the overlapping and uniquity of these genes. **B** Bray–Curtis clustering of strains in feces. **C** relative abundance of top 10 genera of microbiota in feces. **D** Heatmap and clustering of the differential enriched strains of bacteria. **E** Metastats PCA analysis of specific strains in feces of four groups. **F** the relative abundance of *Prevotella* in feces of four groups. **G** the relative abundance of *Akkermansia* in feces of four groups. **H** Metastats analysis of top 12 genera of microbiota in feces of four groups. **I** correlation analysis of top-5-changed abundant genera, top-5-changed differential metabolites and top-5-changed lipid

Accumulating evidence highlights the role of gut microbiota in berberine's effects, given its poor solubility and pharmacokinetics. Metagenomic sequencing of mice feces revealed reduced non-redundant gene counts in berberine-prevention and berberine-treatment groups (Fig. 4A), consistent with berberine's antibiotic-like properties. Bray–Curtis clustering showed distinct microbiota compositions in ND and HFD groups, with berberine groups clustering between them (Fig. 4B). PCA confirmed unique clustering patterns among the four groups (Fig. 4C&D).

Analysis of the top 10 abundant genera (Fig. 4E) showed elevated Prevotella in the HFD group, reduced by berberine (Fig. 4F&G), while *Akkermansia*, significantly depleted in HFD mice, was restored by berberine treatment but less affected by prevention (Fig. 4G). The abundance of *Akkermansia* positively correlated with plasma cholic acid, a cholesterol-derived metabolite critical for gut microbiota regulation (Fig. 4I). These findings highlight *Akkermansia* as a key responder to berberine, influencing systemic metabolism.

### The lipid-reduction effect of berberine is enhanced by *Akkermansia*

**Fig. 5:**
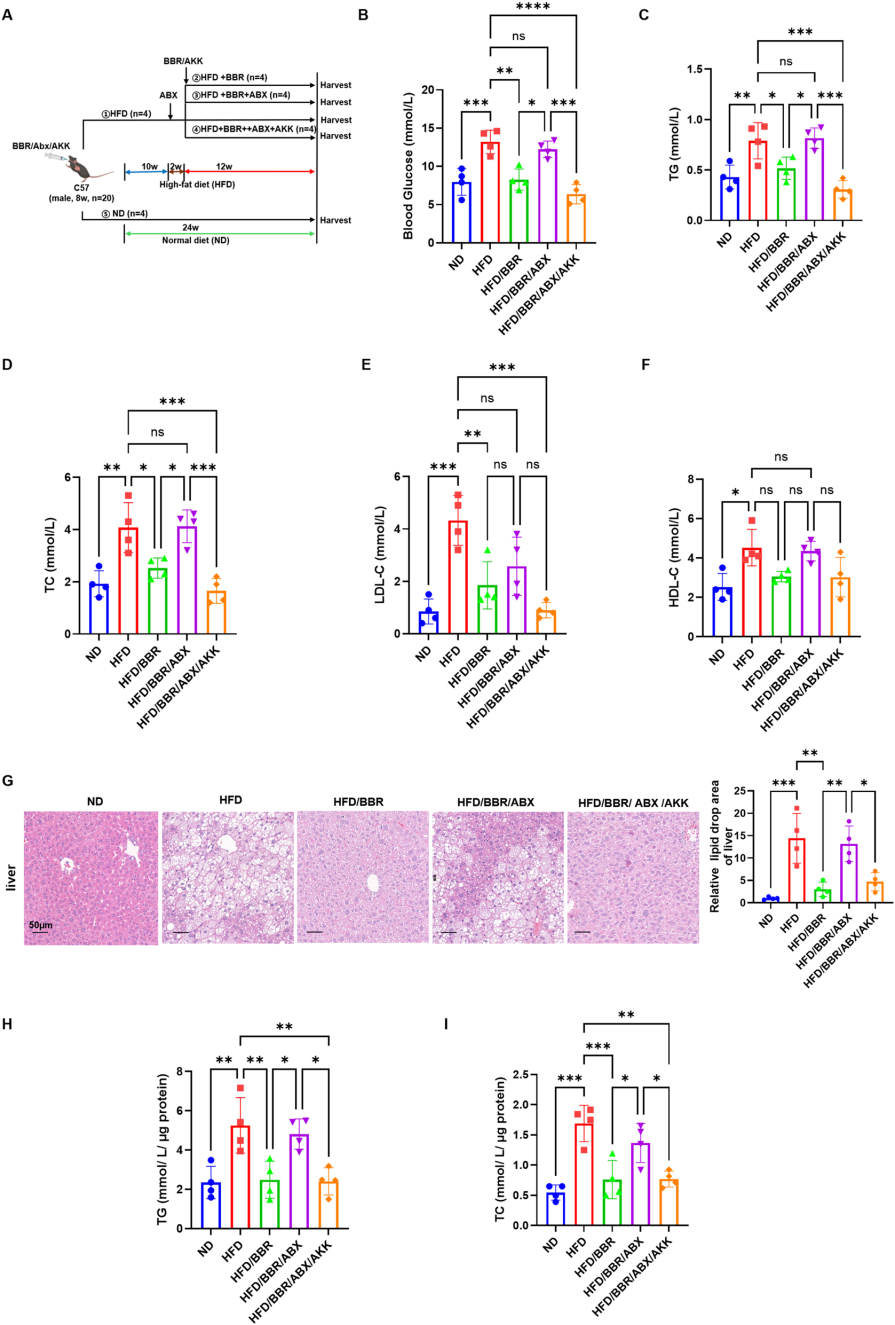
Depletion of *Akkermansia* attenuated the protective effect of berberine. **A** animal models of antibiotic cocktail (ABX) and *Akkermansia* administrated. **B** plasma concentration of blood glucose in different groups of mice after overnight fasting. **C** plasma concentration of total triglyceride in different groups of mice after overnight fasting. **D** plasma concentration of total cholesterol in different groups of mice after overnight fasting. **E** plasma concentration of LDL-C in different groups of mice after overnight fasting. **F** plasma concentration of HDL-C in different groups of mice after overnight fasting. **G** representative image of H&E staining of hepatic tissue in different groups. **H** TG quantification in hepatic tissue. **I** TC quantification in hepatic tissue. These results are presented as mean ± SD (biological replicates, *n* = 4 for liver assays and *n* = 4 for serum assays). (*P < 0.05, **P < 0.01, ***P < 0.001, all the data significance was analyzed by ANOVA by Graph Pad Prism Software V.9.0). These experiments were repeated at least three times

*Akkermansia*, a probiotic residing in the intestinal mucus layer, plays a crucial role in berberine's lipid-reduction effects [18]. Gut microbiota depletion with antibiotics (ABX) [19] (Fig. 5A) diminished berberine's metabolic benefits, increasing glucose, triglycerides, cholesterol, and LDL-C levels while exacerbating hepatic steatosis (Fig. 5B-I). Transplanting *Akkermansia* restored berberine's protective effects (Fig. 5B-I).

**Fig. 6:**
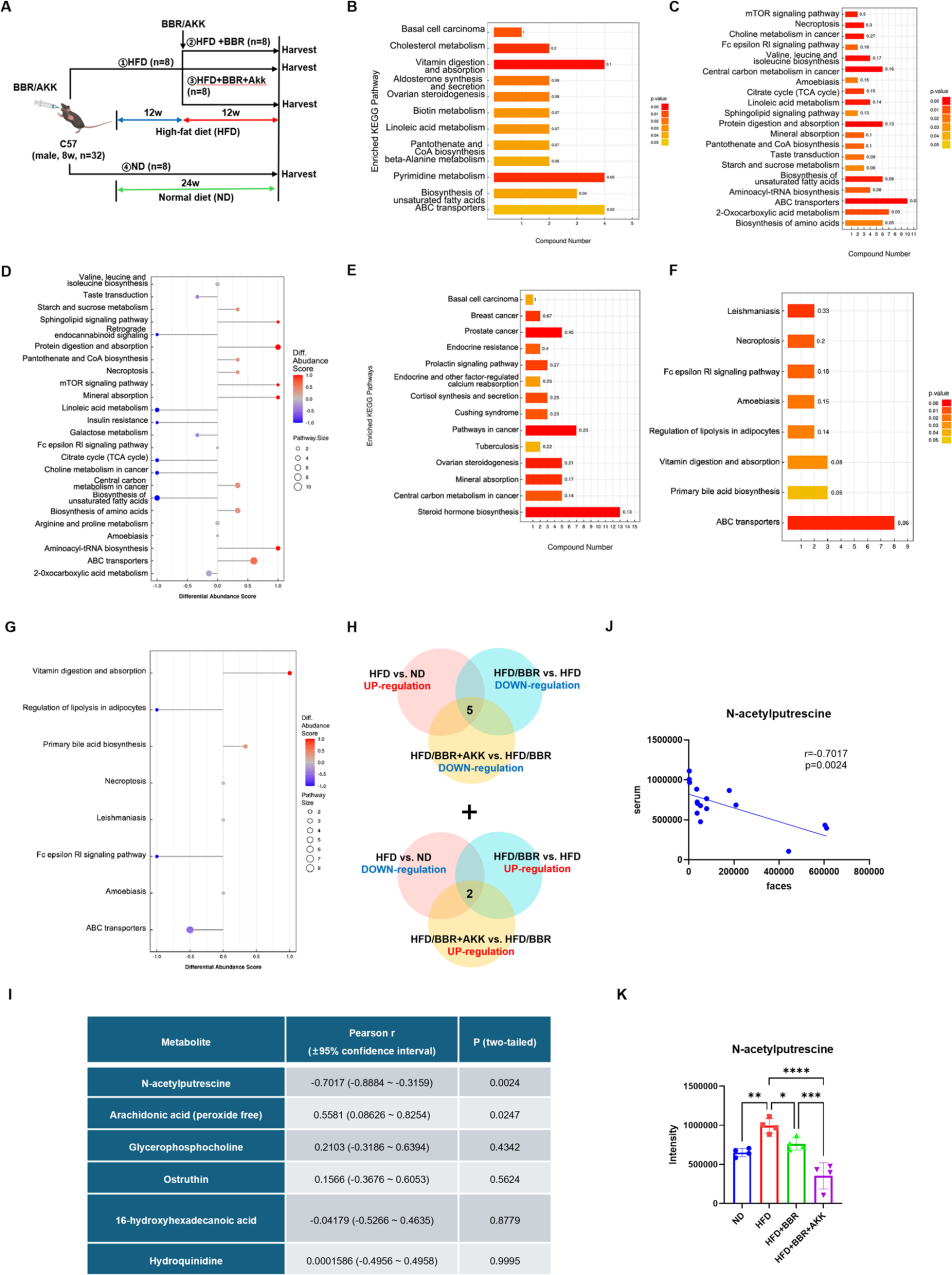
Metabolomics analysis of plasma and feces of berberine and *Akkermansia* gavage mice. **A** Animal models of *Akkermansia* and berberine co-treatment. **B**–**D** KEGG enrichment and differential abundance score of different groups (**B**. ND vs. HFD; **C**. HFD/BBR + AKK vs. HFD; **D**. differential abundance score of HFD/BBR + AKK vs. HFD) in plasma. **E**–**G** KEGG enrichment and differential abundance score of different groups (**E**. ND vs. HFD; **F**. HFD/BBR + AKK vs. HFD; **G**. differential abundance score of HFD/BBR + AKK vs. HFD) in feces. **H** Venn diagram of differential expressed metabolites that possibly affected by berberine and *Akkermansia*. **I** correlation of metabolites in feces and plasma. **J** dot blot of the intensity of N-acetylputrescine in feces and plasma, and the regression curve of intensity in feces and plasma. **K** the intensity of N-acetylputrescine in serum. These results are presented as mean ± SD (biological replicates, n = 5 for metabonomics). (*P < 0.05, **P < 0.01, ***P < 0.001, ****P < 0.0001, all the data significance was analyzed by ANOVA by Graph Pad Prism Software V.9.0). These experiments were repeated at least three times

In mice gavaged with berberine and *Akkermansia* after 3 months of HFD (Fig. 6A), combined treatment further reduced glucose, triglycerides, cholesterol, and hepatic TG and TC compared to berberine alone, with improved hepatic steatosis (Fig. S4). Metabolomics analysis showed altered plasma and fecal metabolites, reduced unsaturated fatty acid biosynthesis, and enhanced aminoacyl-tRNA biosynthesis and bile acid metabolism (Fig. 6B-G). These findings align with a positive correlation between *Akkermansia* abundance and cholic acid (Fig. 4I).

**Fig. 7:**
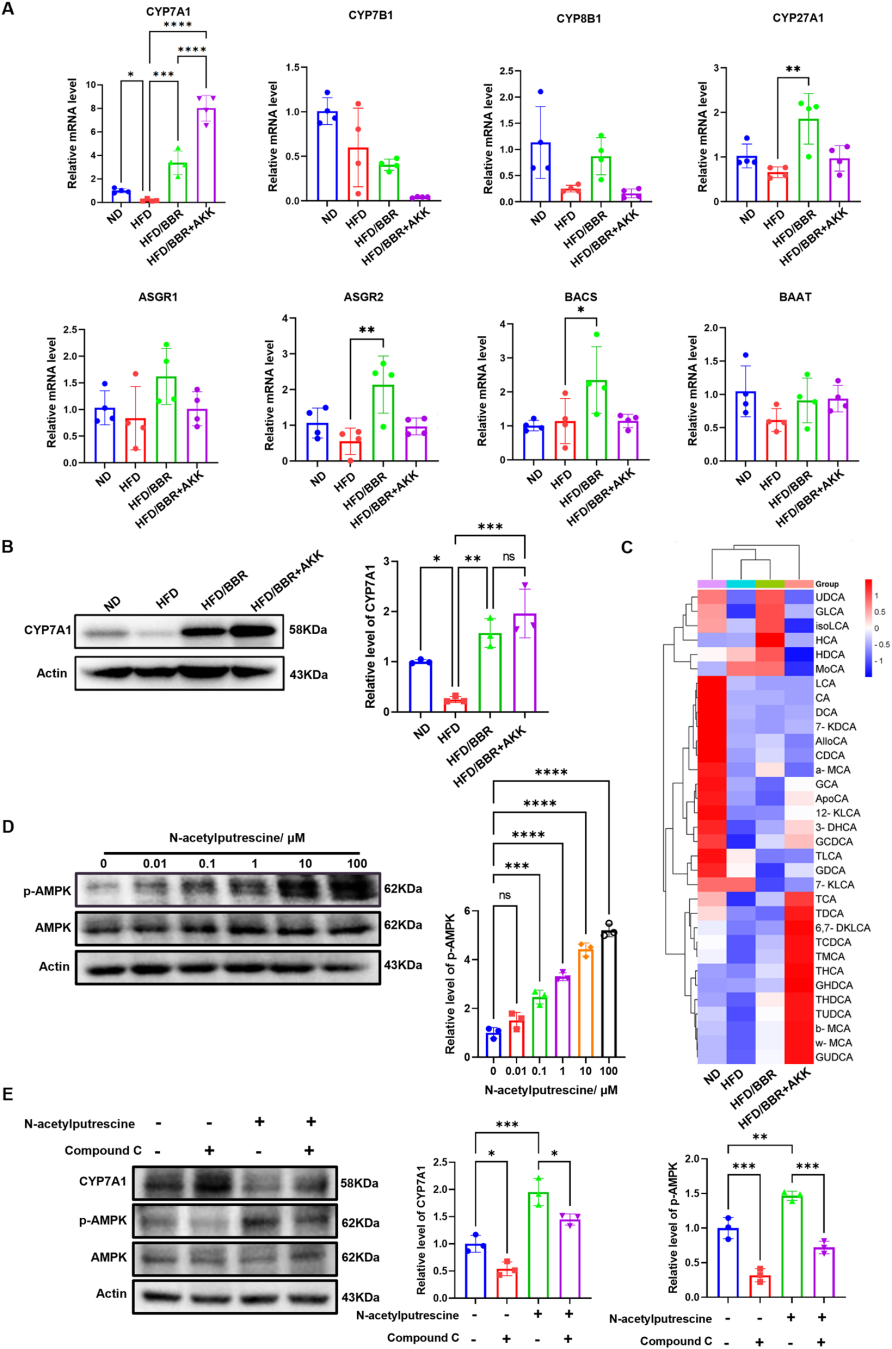
Berberine and *Akkermansia* promotes bile acid biosynthesis in liver by promoting CYP7A1. **A** relative mRNA level of cholesterol metabolism genes in mice liver. **B** representative western blot of CYP7A1 in mice liver of different groups. **C** targeted metabolomics of bile acid in liver and the clustering. **D** representative western blot of phosphorylation of AMPK in AML12 cell treated by N-acetylputrescine. These results are presented as mean ± SD (biological replicates, *n* = 5 for WB; *n* = 4 for Realtime PCR). (**E**) representative western blot of CYP7A1 and phosphorylation of AMPK in AML12 cell treated by N-acetylputrescine and compound C (*P < 0.05, **P < 0.01, ***P < 0.001, ****P < 0.0001, all the data significance was analyzed by ANOVA by Graph Pad Prism Software V.9.0). These experiments were repeated at least three times

In fact, we also found berberine could promote the expression of CYP7A1, the key and first enzyme in transforming cholesterol into bile acid [20]. Berberine enhanced CYP7A1 expression, converting cholesterol to bile acids, an effect amplified by *Akkermansia* (Fig. 7A&B). Targeted metabolomics of bile acids in the liver showed that berberine alone had a minimal effect on bile acid concentration, but its combination with *Akkermansia* significantly increased bile acid levels in the liver (Fig. 7C).

To investigate the potential metabolites involved in the lipid-lowering effects of berberine and *Akkermansia*, differential metabolites in serum were first analyzed between the HFD vs. ND groups, HFD/BBR vs. HFD groups, and HFD/BBR + AKK vs. HFD groups. Five metabolites were found to be increased in the HFD group and decreased after BBR and *Akkermansia* treatment, while two metabolites were decreased in the HFD group and increased after BBR and *Akkermansia* treatment (Fig. 6H). Among these seven metabolites, six were shared between serum and feces. A correlation analysis was performed between serum and fecal metabolite abundances (Fig. 6J). Notably, N-acetylputrescine exhibited a strong negative correlation between serum and fecal metabolites (Fig. 6I&J). In serum, the abundance of N-acetylputrescine was significantly increased in the HFD group, significantly reduced after BBR treatment, and further decreased after the combined treatment of BBR and *Akkermansia* (Fig. 6K, Fig. S5).

The AMPK signaling pathway is a key mechanism through which berberine exerts its lipid-lowering effects [21] and negatively regulates the expression of CYP7A1 [22]. Interestingly, N-acetylputrescine increased the phosphorylation level of AMPK in AML12 hepatocytes in a concentration-dependent manner (Fig. 7D). Additionally, N-acetylputrescine downregulated CYP7A1 protein expression in AML12 hepatocytes (Fig. 7E), while the AMPK inhibitor (Compound C, 10 μM) ameliorated N-acetylputrescine-induced CYP7A1 protein expression (Fig. 7E).

### Berberine restores gut barrier function by preserving epithelial tight junction

**Fig. 8:**
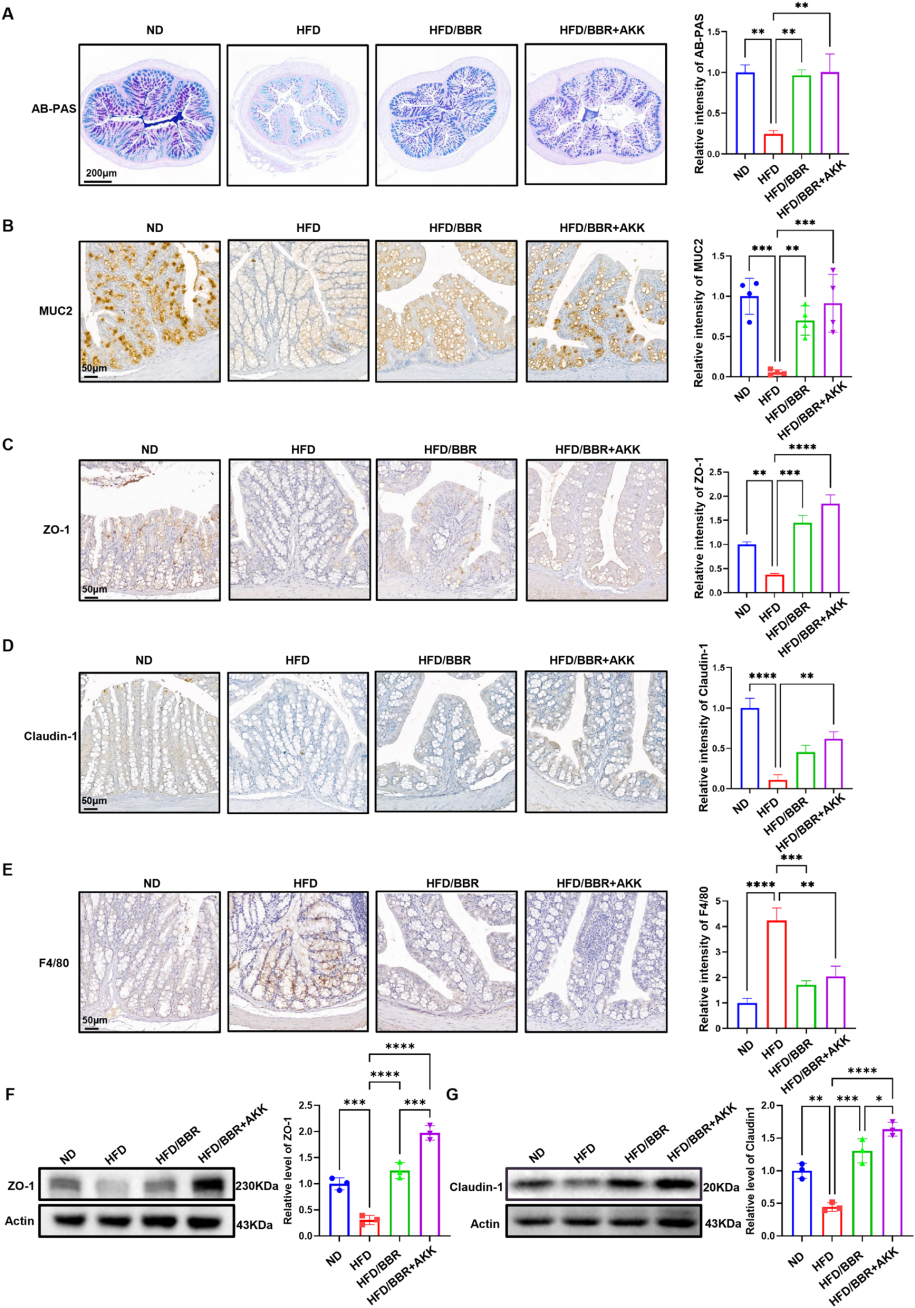
Berberine preserves intestinal mucus layer and tight conjunction. **A** representative image of AB-PAS staining of colon in mice receiving HFD, HFD/BBR and HFD/BBR + AKK, and the statical analysis of the intensity. **B** representative image of immunochemistry staining of MUC2 in the colon. **C** representative image of immunochemistry staining of ZO-1 in the colon. **D** representative image of immunochemistry staining of Claudin-1 in the colon. **E** representative image of immunochemistry staining of F4/80 in the colon. **F** representative western blot of ZO-1 in the colon. **G** representative western blot of claudin-1 in the colon. These results are presented as mean ± SD (biological replicates, *n* = 4 for IHC staining; *n* = 5 for WB). (*P < 0.05, **P < 0.01, ***P < 0.001, ****P < 0.0001, all the data significance was analyzed by ANOVA by Graph Pad Prism Software V.9.0). These experiments were repeated at least three times

HFD impairs gut barrier function, reducing goblet cells, mucus layer thickness, and MUC-2 which is responsible for the production of intestinal mucus layer [23] (Fig. 8A&B). Berberine administration preserved MUC-2 and improved the expression of tight junction proteins, including claudin-1 and ZO-1 [24], which were significantly decreased by HFD (Fig. 8C&D, F&G). Co-administration of berberine and *Akkermansia* further enhanced ZO-1 and claudin-1 expression and reduced macrophage infiltration in the colon (Fig. 8E), mitigating HFD-induced inflammation.

These data indicate that berberine targets *Akkermansia* to maintain the mucus layer and intestinal tight junctions, thereby reducing N-acetylputrescine leakage, promoting CYP7A1 protein expression, and improving bile acid metabolism. Furthermore, the combined treatment of berberine and *Akkermansia* can further enhance the intestinal protective effects of berberine.

## Discussion

The distinguished lipid-lowering and metabolic modulation effects of berberine are widely reported in mice and humans. We and other researchers all revealed the protective effect of berberine against diabetic cardiomyopathy [25], diabetic cognitive impairment [26], diabetic nephropathy [27] and diabetic retinopathy [28]. All these evidences support the idea that berberine can be a strong natural product against metabolic disorders and related complications. Data from our study also support such idea. In our study, we found that various kinds of lipids were altered by berberine treatment, among which the most significant modulates lipids metabolism. Recently, placebo-controlled trials revealed that berberine could reduce LDL-C, total cholesterol and triglyceride in human volunteers [29], which was consistent with our findings. Cholesterol-reduction effect of berberine is of important pharmacological significance. We showed that berberine could induce the expression of CYP7A1 and hence increase the consumption of cholesterol. All these data suggest that berberine can be an effective anti-hyperlipidemia agent in the future, besides its anti-diarrhea effect currently [30].

However, one of the unrevealed questions is that the chemical structure of berberine determines its poor solubility, which means that the molecular effect of berberine may be indirect. Recently, several reports have suggested that the strong metabolic modulation effect of berberine is related to the existence of gut microbiota, since germ-free mice receiving berberine showed less protective effects [31]. By applying metagenomics and analyzing differential expressed microbial strains, we found that *Akkermansia* was the most significantly altered colony after berberine treatment. Meanwhile, several reports also indicated that *Akkermansia* is the key gut microbiota that determines the protective effect of berberine [32].

*Akkermansia* is thought to be a probiotic which can resist high-fat diet induced metabolism disorder [33]. Previous reports suggest that the abundance of *Akkermansia* correlates with the mucus layer thickness [34]. As an important component of the gut barrier, mucus, along with the intestinal tight junction prevents the leakage of toxins in the gut duct to circulation [35]. It is already widely accepted that a high-fat diet significantly impairs gut barrier function and causes elevation of LPS and pro-inflammatory cytokines [36]. We found that berberine combined with *Akkermansia* gavage could preserve the thickness of the mucus layer, the expression of tight junction molecules, and reduce the infiltration of inflammatory macrophages. One of the possible mechanisms that berberine promotes the growth of *Akkermansia* is the enhanced production of polyamines in the gut duct [37], which were also found in untargeted metabolomics of feces of berberine alone with *Akkermansia* gavage mice. In our work, we indeed found that berberine could increase the amount of polyamine and polypeptide in feces. Although we did not incubate these polyamine or polypeptides with microbiota, it is possible that these small molecules provide carbon and nitrogen supply to microbiota to support their growth [38].

Although various studies have already indicated that *Akkermansia* is a kind of probiotics, and berberine could indirectly promote *Akkermansia* growth, the underlying molecular mechanism and metabolic crosstalk between berberine and *Akkermansia* under a high-fat diet is still unrevealed. We first conducted correlation analysis between significantly altered metabolites and lipids and found that the abundance of *Akkermansia* significantly correlated with the plasma level of cholic acids. It was reported that *Akkermansia* could modulate bile acid metabolism [39], while gut bile acids significantly altered the components of gut microflora [40]. Meanwhile, cholic acids is a product of cholesterol, which is catalyzed by cytochrome P450 family. We then consider that the coordinate cholesterol-reduction effect of berberine and *Akkermansia* may be related with boosted cholesterol metabolism. Indeed, CYP7A1 was elevated by berberine, while berberine alone with *Akkermansia* gavage further promoted its expression. This may explain the significant cholesterol reduction in the liver as well as in plasma. Although ASGR1 was recently reported to be an important cholesterol efflux receptor [41], we did not detect any changes in ASGR1, suggesting that the reduction of cholesterol was more related to increased consumption rather than more secretion. Studies have shown that N-acetylputrescine can promote the growth of *Akkermansia *[42], while berberine administration significantly increases fecal levels of N-acetylputrescine. This may further support the notion that berberine promotes *Akkermansia* growth through an indirect mechanism. Knockouting key enzymes of cholesterol metabolism could provide more compelling evidence. But, in our study, we were unable to define the source of the increased cholic acid and lacked the target cells for knock technology. Therefore, in the next step, we try to identify the target cells and combine with knock technology to elucidate how berberine could modulate bile acid metabolism via prospering gut *Akkermansia*.

Another new finding of our work is that by coordinately analyzing metabolomics changes of feces and plasma of mice, we found several key metabolites that may link the metabolic modulation effect of berberine and *Akkermansia*, while N-acetylputrescine showed the powerful negative correlation between feces and plasma. Serum N-acetylputrescine was increased in HFD mice, whereas its levels were reduced after berberine treatment and further decreased with the combined treatment of *Akkermansia*. N-acetylputrescine is a polyamine and an important gut microbiota-derived metabolite [43, 44], which leaks into the circulation when the intestinal barrier is compromised [45]. High-fat diets have been reported to impair intestinal barrier function, and according to our study, both berberine and *Akkermansia* exhibited significant protective effects on intestinal barrier integrity. Therefore, we propose that berberine and *Akkermansia* reduce the intestinal leakage of N-acetylputrescine by restoring the intestinal barrier integrity disrupted by HFD. Further studies revealed that N-acetylputrescine inhibits bile acid metabolism by reducing CYP7A1 expression in the liver. This effectively explains the powerful correlation between *Akkermansia* and bile acids observed in the multi-omics analysis.

Although we analyzed the metabolic changes of berberine-administrated mice and indicated that *Akkermansia* was the key gut microbe that coordinates berberine’s metabolic modulation effect, our study still has limitations. Utilizing knockdown techniques to elucidate the specific metabolic pathways underlying the actions of berberine and Akkermansia holds highly valuable and the lack of this is the greatest regret of our study. Future research can apply them to investigate the precise strain of gut microbe influenced by berberine.

These findings highlight the potential of berberine as a therapeutic agent for metabolic disorders, with *Akkermansia* as its target, reducing N-acetylputrescine leakage by maintaining intestinal integrity. We also identified that serum N-acetylputrescine can interfere with AMPK and CYP7A1 to regulate the ability to transform cholesterol. Our work replenishes the pharmacological mechanism of berberine and indicates combination with *Akkermansia* can ameliorate hyperlipidemia more effectively.

## Materials and methods

### High-fat diet-induced obesity mice model and animal treatment.

A total of 140 male C57Bl/6 mice weighing between 20 and 25 g (aged 8–9 weeks) were obtained from Vital River Laboratory Animal Technology Co., LTD., and the mice were housed under strict specific-pathogen-free conditions. The animal facility maintained a controlled environment with a 12-h light/dark cycle, ambient temperature of 22 ± 1 °C, and relative humidity of 50 ± 10%. All mice were allowed ad libitum access to autoclaved chow and sterile water. The mice were randomly assigned to receive either a standard chow diet (XTC01WC-001, Xietong Bio, Nanjing, China, which contains 21.3% protein, 67.3% carbohydrate, 11.4% fat) or a high-fat diet (XTM04-001, Xietong Bio, Nanjing, China, which contains 20% protein, 20% carbohydrate, 60% fat).

In the pre-experiment, mice were divided into six groups (n = 8) after one week of adaptation: normal diet (ND), high-fat diet (HFD), and HFD groups treated with berberine (HY-18258, MedChemExpress, Shanghai, China) at low (10 mg/kg/day), medium (25 mg/kg/day), or high dosage (50 mg/kg/day), as well as a metformin (5 mg/kg/day) group as a positive control.

After confirming that 25 mg/kg/day was the effective dosage, the main experiment began. Mice were divided into five groups (n = 8): ND, HFD, berberine gavage with HFD, berberine prevention with HFD, and berberine treatment with HFD. The study ran for six months, with weekly body weight monitoring.

Animal models of antibiotic cocktail (ABX) and *Akkermansia* administrated: After 10 weeks of HFD feeding, an ABX was administered to deplete the gut microbiota. After 12 weeks of HFD feeding, the mice received berberine in conjunction with *Akkermansia*. Mice were divided into five groups (n = 4): ND, HFD, HFD + berberine, HFD + berberinre + ABX, HFD + berberine + ABX + *akkermansia*. Gut microbiota was depleted in some mice using an ABX of streptomycin (5 mg/ml), ampicillin (1 mg/ml), and colistin (1 mg/ml) administered in drinking water for 14 days after 10 weeks of HFD feeding. This was followed by a one-week-on, one-week-off regimen. Mice were gavaged daily with 10⁸ CFU of *Akkermansia* (B161463, Mingzhou biotechnology, Ningbo, China) and 25 mg/kg/day of berberine starting at week 14 for three months. *Akkermansia* was cultured under anaerobic conditions at 37 °C in chopped meat carbohydrate broth, lyophilized, and stored at 4 °C using skim milk as a protective agent.

Animal models of *Akkermansia* and berberine co-treatment. After 12 weeks of HFD feeding, the mice were administered berberine or a combination of berberine and *Akkermansia*. Mice were divided into four groups (n = 8): ND, HFD, HFD + berberine, HFD + berberine + *akkermansia*.

At the end of the experiment, mice were fasted overnight and then euthanized by an intraperitoneal injection of an overdose of pentobarbital sodium. Tissues, including liver and adipose tissues, were fixed in paraformaldehyde, while plasma was collected by EDTA-Na2 anticoagulation and centrifugation. Fecal samples were collected from the colon under sterile conditions for analysis.

### Biochemical analysis of mice plasma

Plasma concentration of glucose, total triglyceride (TG), total cholesterol (TC), LDL-C, HDL-C and GOT was quantified by commercial-available kit (Nanjing JianCheng Biotechnology, Nanjing, China) according to the protocol of manufacturer. Similarly, hepatic level of TG and TC were measured after homogenizing hepatic tissue with 0.1% NP-40 in PBS, and quantified the level of TG and TC according to protein concentration quantified by BCA kit (20201ES, Yeasan, Shanghai, China).

### Histological staining and analyzing

After fixation and embedding, tissue sections were dewaxed, dehydrogenized, and stained using an HE staining kit (G1076, ServiceBio, Wuhan, China) or AB-PAS staining by ServiceBio. Immunohistological staining was performed after permeabilization and blocking with 5% BSA. Primary antibodies used included anti-Claudin1 (A21971, Abclonal, Wuhan, China), anti-ZO-1 (21,773–1-AP, Proteintech, Wuhan,China), anti-MUC2 (27,675–1-AP, Proteintech, Wuhan, China), and anti-F4/80 (29,414-1-AP, Proteintech, Wuhan, China), following manufacturers' dilutions. Sections were incubated with primary antibodies overnight at 4 °C, washed, and treated with HRP-conjugated secondary antibody (AS014, Abclonal, Wuhan, China) at 1:200 for 2 h at room temperature. HRP signals were detected using a DAB staining kit (ServiceBio), scanned with CaseViewer, and analyzed in ImageJ.

### Chemicals and reagents

Berberine (HY-18258, MedChemExpress, Shanghai, China), metformin (HY-B0627, MedChemExpress, Shanghai, China), BSA (GC305010, Servicebio, Wuhan, China), Linoleic acid (HY-N0729, MedChemExpress, Shanghai, China).

### Metabolomics and lipidomics analysis of mice plasma

For metabolomics, 100 μL plasma or 100 mg feces was resuspended in prechilled 80% methanol, incubated on ice, and centrifuged at 15,000 g, 4 °C for 20 min. Supernatant was diluted with LC–MS-grade water to 53% methanol, centrifuged again, and injected into the LC–MS/MS system, performed by Novogene, Tianjin, China.

For lipidomics, 100 μL plasma was mixed with methanol, MTBE, and MS-grade water to induce phase separation. The organic phase was collected, re-extracted, dried, dissolved in isopropanol, and analyzed by LC–MS/MS.

Data analysis, including PCA, PLS-DA, and t-tests, was performed with metaX. Differential metabolites were identified using VIP > 1, P-value < 0.05, and fold change (FC ≥ 2 or ≤ 0.5). Volcano plots and heatmaps were created using the online platform Bioinformatics.com.cn (accessed July 10, 2023).

*PCA* To assess the overall distribution pattern of samples, the degree of separation between groups, and to identify potential outliers, this study employed unsupervised Principal Component Analysis (PCA). PCA reduces the dimensionality of complex datasets through a linear transformation that projects the original variables into a new set of orthogonal axes, known as Principal Components (PCs). The results were visualized using a score plot and a loading plot. The score plot displays the natural clustering or separation of samples, revealing the primary sources of variation between groups. The loading plot identifies the original variables (e.g., metabolites or genes) that contribute most to the observed separation in the score plot, which are considered potential key biomarkers. The quality of the PCA model was evaluated by the cumulative variance contribution rate, typically reported for the first two principal components (PC1 and PC2).

*Correlation Analysis* To investigate linear or monotonic relationships between specific variable pairs (e.g., microbial abundance and metabolite concentration, gene expression and clinical phenotypes), this study utilized Pearson correlation analysis and Spearman's rank correlation analysis, respectively. Pearson correlation was applied for continuous variables that followed a normal distribution, while Spearman's correlation, a more robust non-parametric method, was used for variables that violated the normality assumption or were ordinal. The calculated correlation coefficient quantified the strength and direction of the relationship, and its statistical significance was assessed via a two-tailed t-test. To control the False Discovery Rate (FDR) arising from multiple hypothesis testing, all calculated p-values were adjusted using the Benjamini–Hochberg procedure. An adjusted FDR < 0.05 was considered statistically significant. The correlation results were typically presented as a correlation heatmap.

*Differential Analysis* To identify variables that exhibited significant changes under different experimental conditions (e.g., disease group vs. control group), this study performed differential analysis. For normally distributed variables, Student’s T-test (for two-group comparisons) or one-way ANOVA (for multi-group comparisons) was employed. For non-normally distributed data, the non-parametric Mann–Whitney U test (for two groups) or the Kruskal–Wallis H test (for multiple groups) was used instead. Similarly, the resulting p-values were subjected to FDR correction. Variables with an FDR < 0.05 and an absolute log2-transformed fold change (|log2FC|) greater than 1 were defined as significantly differentially abundant or expressed.

*Functional Enrichment and Pathway Analysis* To biologically interpret the list of significantly differentially expressed genes or metabolites, functional enrichment and pathway analysis were conducted. This study utilized the Kyoto Encyclopedia of Genes and Genomes (KEGG) and Gene Ontology (GO) databases. Enrichment analysis was performed using the hypergeometric distribution test to identify over-represented pathways or functional terms. A p-value < 0.05 was set as the threshold for significant enrichment. This analysis aimed to translate the simple list of differential molecules into biologically meaningful pathways or functional modules, thereby revealing the underlying biological mechanisms.

### High-performance liquid chromatography (HPLC)

HPLC analysis was supported by Servicebio (Wuhan, China) following the 2015 Chinese Pharmacopoeia protocol. Natural medicinal herbs were powdered, and 0.5 g was mixed with 25 mL methanol in a 50 mL tube. After ultrasonic treatment (350W, 35 kHz) for 10 min and overnight incubation, the process was repeated, with methanol added to compensate for weight loss. The mixture was filtered with a 0.22 μm filter, diluted (100 μL to 2000 μL), and 10 μL was injected for analysis.

Chromatographic conditions included a Welch Ultimate Plus C18 column (250 × 4.6 mm, 5 μm) with a DAD detector at 280 nm, a 1 mL/min flow rate, 30 °C column temperature, and a mobile phase of 0.1% phosphoric acid (A) and acetonitrile (B) with gradient elution.

### Gradient Elution

**Table:** 

Time/ min	%A	%B
0	**80%**	**20%**
10	**10%**	**90%**
15	**10%**	**90%**
15.1	**80%**	**20%**
20	**80%**	**20%**

### Metagenomics sequencing and data analysis

Genomic DNA from feces was extracted using a commercial kit (18820ES, Yeasan, Shanghai, China) and randomly sheared into fragments, quality-controlled with 1% agarose gel. Fragments were end-repaired, A-tailed, ligated with Illumina adapters, PCR-amplified, size-selected, and purified. Libraries were quantified with Qubit, real-time PCR, and bioanalyzer, then pooled and sequenced on Illumina platforms based on library concentration and required data.

Raw data were cleaned with Readfq and assembled using MEGAHIT (v1.0.4-beta). DIAMOND software blasted unigenes against bacterial, fungal, archaeal, and viral databases. Krona was used for taxonomic analysis, and the most abundant species from the top 10 was selected for correlation analysis. Analyses were supported by Novogene.

### Western blot and real-time polymerase chain reaction

Intestinal and hepatic tissues were quickly frozen in liquid nitrogen and homogenized in RIPA buffer with protease and phosphatase inhibitors (20138ES and 20109ES, Yeasan, Shanghai, China). After centrifugation (4 °C, 16,000 g, 15 min), the supernatant was collected and quantified using a BCA kit (20201ES, Yeasan, Shanghai, China). Proteins were boiled and analyzed by western blot using a quick western blot kit (20325ES, Yeasan, Shanghai, China) and primary antibodies diluted as per protocols. Primary antibodies used included anti-Claudin1 (A21971, Abclonal, Wuhan, China), anti-ZO-1 (21,773–1-AP, Proteintech, Wuhan, China), CYP7A1 (A22897, Abclonal, Wuhan, China), p-AMPK (AP1441, Abclonal, Wuhan, China), AMPK (A12718, Abclonal, Wuhan, China), Actin (AC026, Abclonal, Wuhan, China).

RNA was extracted with RNA-easy isolation reagent (R701, Vazyme, Nanjing, China), reverse transcribed into cDNA using a commercial kit (RK20429, Abclonal, Wuhan, China), and detected using SYBR Green fast qPCR Mix (RK21203, Abclonal, Wuhan, China).

*CYP27A1*: Forward: CCAGGCACAGGAGAGTACG; Reverse: GGGCAAGTGCAGCACATAG; *CYP7A1*: Forward: GGGATTGCTGTGGTAGTGAGC; Reverse: GGTATGGAATCAACCCGTTGTC; *CYP7B1*: Forward: GGAGCCACGACCCTAGATG; Reverse: TGCCAAGATAAGGAAGCCAAC; *CYP8B1*: Forward: CCTCTGGACAAGGGTTTTGTG; Reverse: GCACCGTGAAGACATCCCC; *BAAT*: Forward: GGAAACCTGTTAGTTCTCAGGC; Reverse: GTGGACCCCCATATAGTCTCC; *BACS*: Forward: GTTCTCCCGTCCAAGACCATT; Reverse: GCTCCGTACAGAGTGTAGCAAG; *Asgr1*: Forward: TGAGCACCCAGGGAAGTAGT; Reverse: CCATTGCCCCGAAATGCAG; *Asgr2*: Forward: CTGCAAGAAGAGTTTCGGACC; Reverse: GTATGGCGTTTGTGCTACCTC; *Actin*: Forward: GGCTGTATTCCCCTCCATCG; Reverse: CCAGTTGGTAACAATGCCATGT. The relative mRNA level was calculated by the 2^−ΔΔCt^ method.

### Statical analysis

Statistical analysis was performed by a researcher who was blinded. Statistical analyzes were performed using Graph Pad Prism Software V.9.0 (San Diego, CA) or bioinformatics online website. Statistical significance was calculated using a one-way analysis of variance (ANOVA). Values of *p < 0.05, **p < 0.01, ***p < 0.001 and ****p < 0.0001 were considered significant between different groups. All the experiments were repeated for at least three times.

The sample size was estimated using G*Power software (version 3.1). With the parameters set at α = 0.05, power (1-β) = 0.8, and based on the effect size derived from preliminary experiments or prior studies, the required independent sample size per group was determined to be n = 4.

## Supplementary Information

Supplementary file 1.

Supplementary file 2.

Supplementary file 3.

Supplementary file 4.

Supplementary file 5.

Supplementary file 6.

Supplementary file 7.

## Data Availability

All data generated or analysed during this study are included in this published article and its supplementary information files.

## Abbreviations

AA: Arachidonic acid
ABX: Antibiotic cocktail
AB-PAS: Stain periodic acid Schiff and Alcian blue stain
BBR: Berberine
AKK: Akkermansia
AMPK: Adenosine 5 ‘-monophosphate (AMP)-activated protein kinase
Berberine: Berberine
CYP7A1: Cholesterol 7-alpha hydroxylase
EPA: Eicosapentaenoic acid
FA: Fatty acid
GOT: Glutamic oxaloacetic transaminase
HDL-C: High-density lipoprotein cholesterol
HFD: High fat diet group
KEGG: Kyoto encyclopedia of genes and genomes
LDL-C: Low-density lipoprotein cholesterol
LPS: Lipopolysaccharides
ND: Normal diet group
PC: Phosphatidylethanolamine
PC: Phosphatidylcholine
PCA: Principal component analysis
PE: Phosphatidyl ethanolamine
PI: Phosphatidylinositol
PLS‐DA: Partial least squares discriminant analysis
PS: Phosphatidylserine
SM: Sphingomyelin
SPF: Specific-pathogen-free
TAG: Triacylglycerol
TC: Total cholesterol
TG: Total triglyceride
ZO-1: Zonula occludens protein 1
