# Supplementary material for: Berberine ameliorates high-fat diet-induced metabolic disorders through promoting gut Akkermansia and modulating bile acid metabolism
Source: Chin Med. 2025 Nov 17;20:190. doi: 10.1186/s13020-025-01251-6 (PMC12621396; doi:10.1186/s13020-025-01251-6)
Supplement: Supplementary file 6 [file 13020_2025_1251_MOESM6_ESM.docx]

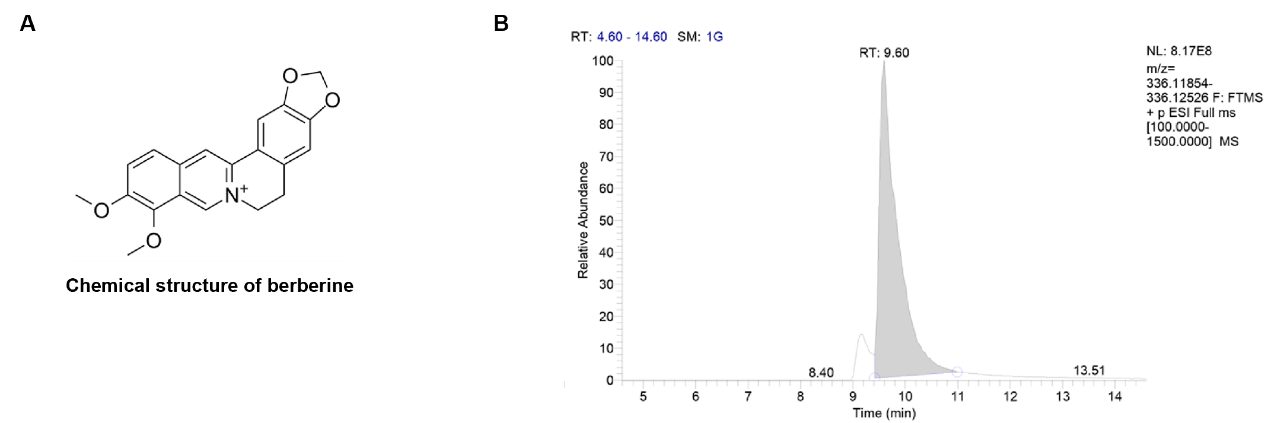


**Fig. S1.** **Berberine is the active intergradient in *Coptidis Rhizoma*.** (A) chemical structure of berberine. (B) specific peak of berberine analyzed by HPLC.


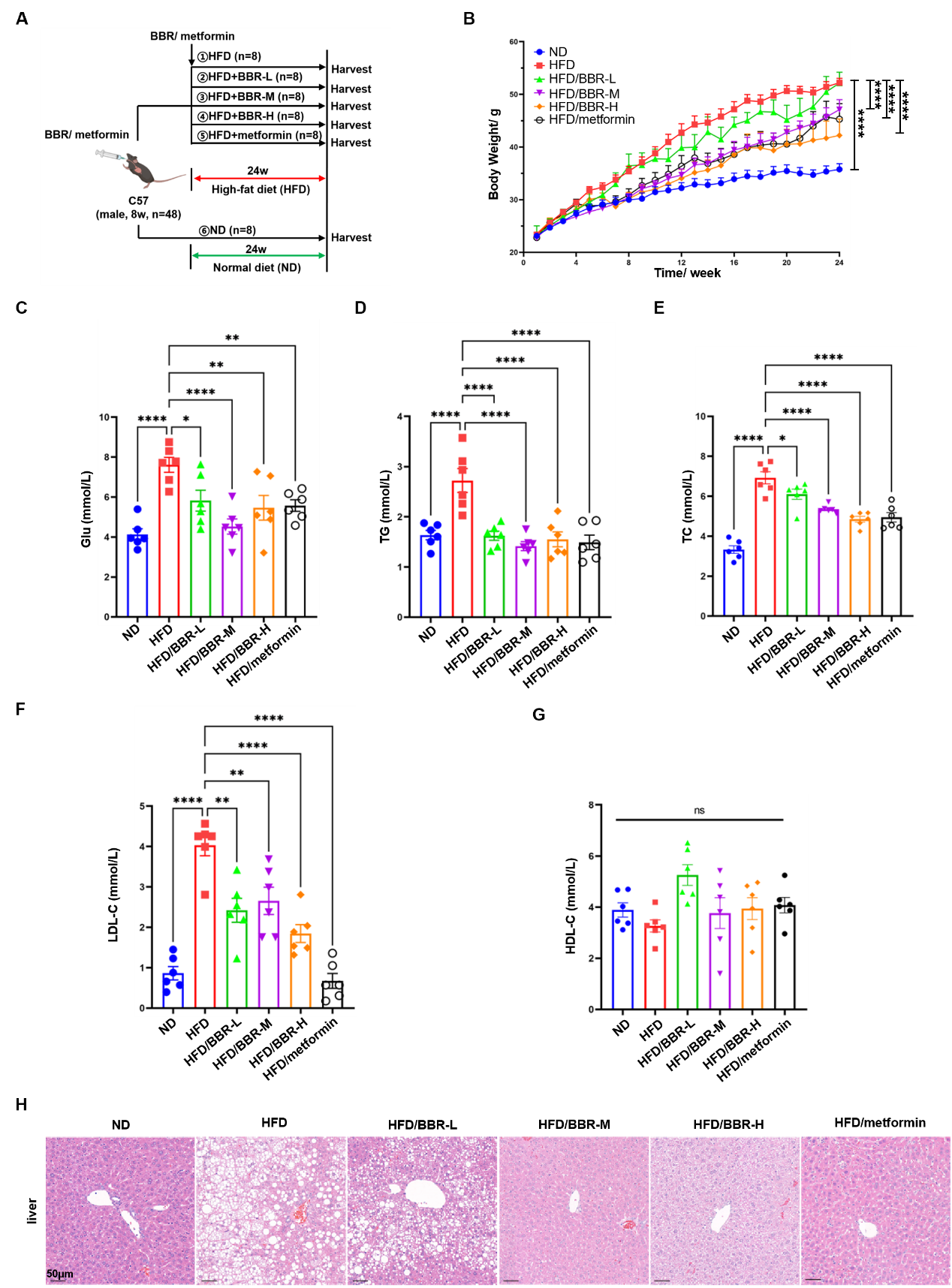


**Fig. S2. Different dosage of berberine ameliorate high-fat diet induced metabolic disorder.** (A) animal model treated with different BBR concentrations and metformin. (B) body weight record of mice in different groups. (C) plasma concentration of blood glucose in different groups of mice after overnight fasting. (D) plasma concentration of total triglyceride in different groups of mice after overnight fasting. (E) plasma concentration of total cholesterol in different groups of mice after overnight fasting. (F) plasma concentration of LDL in different groups of mice after overnight fasting. (G) plasma concentration of HDL in different groups of mice after overnight fasting. (H) representative image of H&E staining of hepatic tissue in different groups.  These results are presented as mean ± SD (biological replicates, *n* = 6 for serum assays). (*P<0.05, **P<0.01, ***P<0.001, ****P<0.0001, all the data significance was analyzed by ANOVA by Graph Pad Prism Software V.9.0)


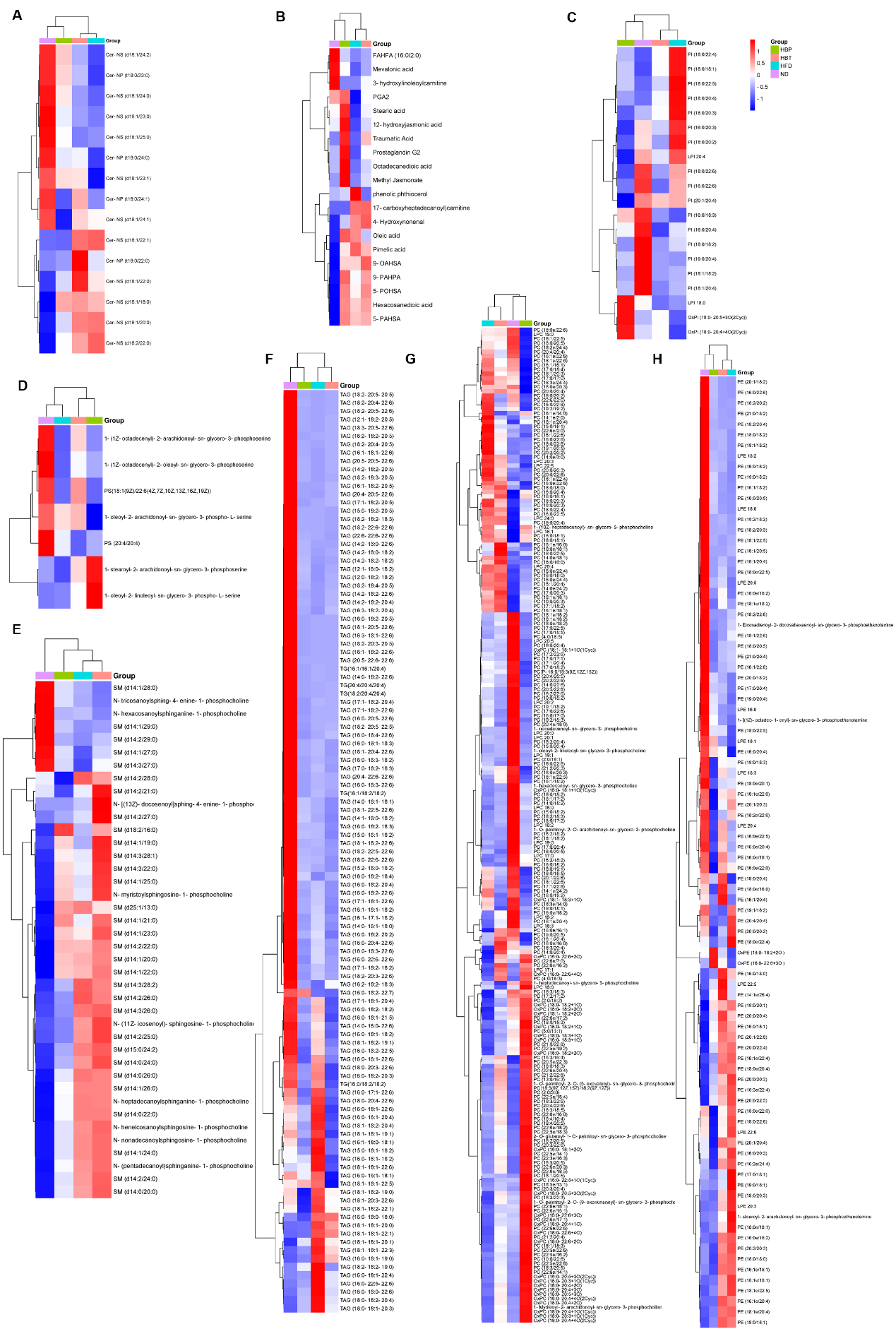


**Fig. S3. Heatmap of different kinds of lipids, including ceramide, FA, PI, PS, SM, PC, TAG and PE.**


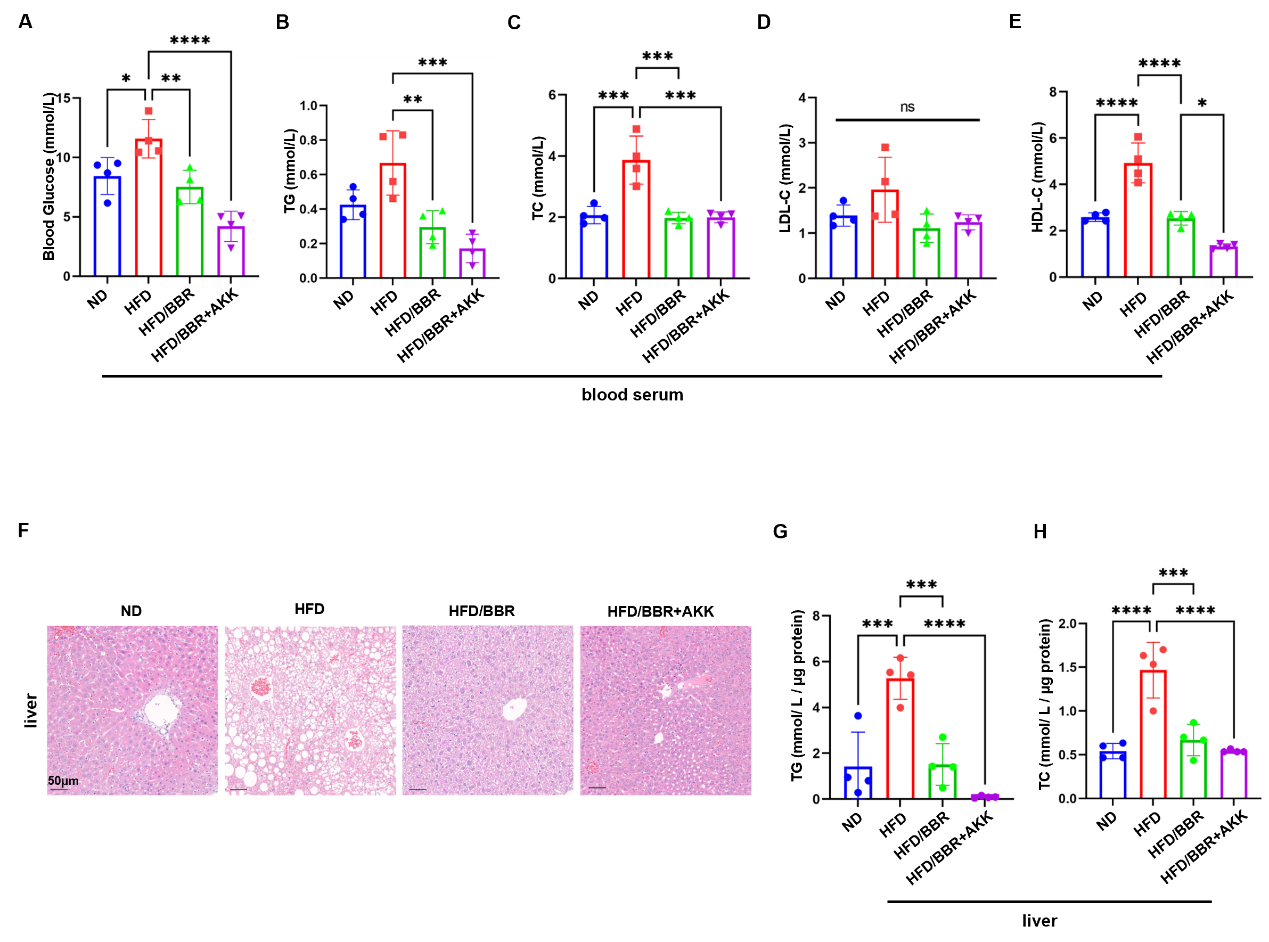
**Fig. S4. *Akkermansia* enhances the lipid reduction effect of berberine.** (A) plasma concentration of blood glucose (B) plasma concentration of total triglyceride. (C) plasma concentration of total cholesterol (D) plasma concentration of LDL. (E) plasma concentration of HDL. (F) representative image of H&E staining of hepatic tissue in five groups. (G) TG quantification in hepatic tissue. (H) TC quantification in hepatic tissue. These results are presented as mean ± SD (biological replicates, *n* = 4 for liver assays and *n* = 4 for serum assays). (*P<0.05, **P<0.01, ***P<0.001, ****P<0.0001, all the data significance was analyzed by ANOVA by Graph Pad Prism Software V.9.0).


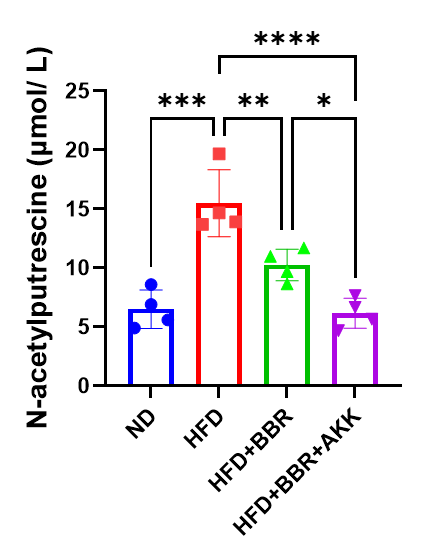


**Fig. S5.**

The concentration of N-acetylputrescine in mouse serum was validated using HPLC. These results are presented as mean ± SD (biological replicates, *n* = 4 for HPLC). (*P<0.05, **P<0.01, ***P<0.001, ****P<0.0001, all the data significance was analyzed by ANOVA by Graph Pad Prism Software V.9.0).
